# Recurrent Vertigo of Childhood: Clinical features and prognosis

**DOI:** 10.3389/fneur.2022.1022395

**Published:** 2022-09-28

**Authors:** Konstanze Dunker, Lutz Schnabel, Eva Grill, Filipp Maximilian Filippopulos, Doreen Huppert

**Affiliations:** ^1^German Center for Vertigo and Balance Disorders (DSGZ), Ludwig-Maximilians-Universität München, Munich, Germany; ^2^Department of Neurology, University Hospital, Ludwig-Maximilians-Universität, Munich, Germany; ^3^Institute for Medical Information Processing, Biometrics and Epidemiology, Ludwig-Maximilians-Universität, Munich, Germany

**Keywords:** Recurrent Vertigo of Childhood, children, adolescents, vertigo, dizziness, vestibular migraine of childhood

## Abstract

**Introduction:**

“Recurrent Vertigo of Childhood” (RVC) has recently replaced the term “Benign Paroxysmal Vertigo of Childhood” and was defined as recurrent spells of vertigo without evidence of a vestibular migraine of childhood (VMC). RVC and VMC are considered the most frequent causes of vertigo and dizziness in children below 18 years of age. Diagnosis might be challenging since clinical features of RVC and VMC may overlap.

**Objective:**

This study aims to characterize clinical and instrument-based findings in patients with RVC and to evaluate the course of the disorder.

**Methods:**

We prospectively collected clinical and instrument-based data of children/adolescents younger than 18 years, who presented at the German Center for Vertigo and Balance Disorders (DSGZ) at the LMU University Hospital in Munich. All patients underwent a comprehensive neurological, ocular motor, vestibular and cochlear examination. Furthermore, findings from follow-up examinations were analyzed.

**Results:**

Overall 42 children (24 male and 18 female) with RVC were included in the study. The mean age at diagnosis was 7 ± 3.6 years with a mean onset of symptoms at the age of 5.6 ± 3.4 years. Attack duration ranged between 1 min and 4 h. The most common accompanying symptoms included nausea, vomiting, expression of fear, and falls. Non-migrainous headaches were reported by 11 patients during initial presentation, 7 of whom were later diagnosed with migraine. Female patients showed a higher age at symptom onset, a higher attack frequency, and attack duration. Eleven of the 24 patients seen at a 3.5 year follow-up reported a complete cessation of attacks. Patients still experiencing vertigo attacks had a significantly reduced attack frequency, especially those who implemented at least one prophylactic measure.

**Conclusion:**

A precise characterization of symptoms is essential for diagnosing children with RVC. Age at symptom onset does not exceed the age of 12. Gender-specific differences should be considered and may further support the evidence of an association with migraine. The disease course of RVC is benign, nevertheless implementing prophylactic measures such as regular exercise, increased fluid intake, sleep hygiene, and relaxation exercises, can improve attack frequency.

## Introduction

Approximately 5% of children and adolescents complain of vertigo/dizziness and balance problems (1). Symptoms can occur with varying frequencies, from only once over a certain time period (monophasic), with recurrent attacks (episodic), or persistently (2). Children commonly suffer from episodic vertigo attacks with the most frequent diagnoses being “Recurrent Vertigo of Childhood” (RVC) and “Vestibular Migraine of Childhood” (VMC) (2–5).

RVC, as recently defined by the Bárány Society, is characterized by at least three episodes with vestibular symptoms of moderate or severe intensity, lasting between 1 min and 72 h without a current or past history of migraine with or without aura and associated migraine features in over 50% of episodes in children and adolescents below 18 years of age (6). The syndrome was first described in 1964 by Basser (7) with an onset at the age of four. It was labeled “Benign Paroxysmal Vertigo of Childhood” due to the spontaneous cessation of attacks between ages 8–10 without persistent vestibular or neurological deficits. Among “dizzy” children and adolescents, RCV is diagnosed in about 18–23% of cases and constitutes the second most frequent diagnosis in patients under the age of 18 (2, 3, 8, 9). Notably, the proportion of children with RCV has been shown to be especially high in children under the age of seven (71–87.5%) and between seven and 12 years (30%) (8, 10, 11). Symptom remission typically occurs between 3 months to 8 years after onset (12, 13), but may persist longer in some children/adolescents or may be followed by the diagnosis of migraine (13, 14). The underlying pathophysiology of RVC is still unknown, but a possible link with migraine has been suggested due to a high reported prevalence of migraine in children suffering from RVC (12, 13, 15, 16).

Diagnosing RVC can be challenging, particularly in its distinction from VMC. Due to the lack of prospective clinical studies on children/adolescents with RVC, the diagnostic criteria of the Bárány Society (6) define RVC as episodes with vestibular symptoms that do not fulfill the criteria of VMC, or any other medical condition. In other words, RVC is a diagnosis by exclusion; inclusion criteria based on clinical or instrument-based findings have not been included. However, a number of studies have described distinct findings in patients with RVC, for example evidence of vestibulo-cochlear dysfunction (15) or elevated serum levels of creatine kinase-MB (CK-MB) (17).

In the present study, in order to characterize children/adolescents with RVC in detail, we prospectively collected clinical and instrument-based findings including ocular motor testing, a broad vestibular and cochlear assessment as well as imaging results. Furthermore, follow-up examinations up to 4 years were conducted in a proportion of patients to better characterize the course and prognosis of the disease.

## Materials and methods

### Subjects

All children and adolescents diagnosed with RVC at the German Center for Vertigo and Balance Disorders (DSGZ) at the LMU University Hospital in Munich between January 2016 and May 2022 were prospectively included in the study. All patients fulfilled the current diagnostic criteria for RVC of the Bárány Society (6). Written informed consent was obtained from all participants included in the study and their parents/legal guardians.

### Clinical and instrument-based examination

All included patients underwent structured history-taking and standardized neurological, ocular motor and neuro-otological examinations. Data collected included age of onset, frequency and duration of attacks, trigger factors, underlying medical conditions, and family history. Furthermore, the following instrument-based examinations were conducted if possible (depending on age):

- Caloric irrigation and video Head-Impulse-Test (vHIT) were used to quantify peripheral vestibular function of the horizontal semicircular canal. Caloric irrigation values above 30% side asymmetry and/or a vHIT gain of less than 0.7 were considered pathological.
- Ocular and cervical Vestibular-Evoked Myogenic Potentials (o- and c-VEMP's) were utilized to evaluate function of the utricle and saccule.
- Posturography was used to quantify body sway patterns.
- Audiometry and Auditory Evoked Potentials (AEP's) were used to evaluate cochlear function.

Follow-up visits were conducted when medically indicated. In addition, each patient was contacted to assess the course of the disease using a standardized questionnaire. The questionnaire included questions about current attack characteristics, accompanying symptoms, trigger factors, implemented prophylactic measures and, if applicable, age of attack cessation. To evaluate a possible link to migraine disorders, we ascertained information about occurrence and characteristics of headaches as well as accompanying symptoms during headache attacks.

### Statistics

After data collection, all data were irreversibly anonymized for data analyses. For data description, we used mean values and standard deviation for continuous variables and absolute and relative frequencies for categorical variables. Statistical differences were calculated between male and female patients, between attack-free patients and patients with ongoing attacks as well as differences between characteristics at first presentation and follow-up. We performed a *t*-test to test for differences in continuous variables and a Pearson's chi-squared test for categorical variables.

## Results

### Patient and attack characteristics

Of 453 patients who presented at the DSGZ during the recruitment phase, 42 patients between 2 and 15 years were diagnosed with RVC. The mean age at first clinical evaluation was 7.0 ± 3.6 years. The mean age when symptoms first were noted by patients, or their parents was 5.6 ± 3.4 years. Female patients had a significantly higher age of symptom onset than male patients (male: 4.7±3.7 years; female: 6.9 ± 3.3 years; *p* = 0.029). Attack frequency varied between one attack per year and three attacks per day with a mean of 13.7 ± 18.9 attacks per month. Attack duration ranged between 1 min and 4 h; female patients experienced significantly longer (mean: male = 13.3 min; female = 41.2 min; *p* = 0.008) and more frequent attacks than male patients (mean: male = 8.8 ± 10.2; female = 20.3 ± 25.3; *p* = 0.042). A clustering of attacks up to seven days, followed by a longer period without attacks was reported in 33% of the patients (Table 1).

**Table 1:** Characteristics of vertigo/imbalance attacks in 42 children with Recurrent Vertigo of Childhood.

**Attack characteristics**		
Vertigo type	Torsional	34 (81%)
	Swaying	15 (36%)
	Dizziness	5 (12%)
	Duration in minutes [*mean±sd; (min.; max)*]	25.5 ± 36.4; [1; 240]
	Attack frequency per month [*mean±sd; (min.; max)*]	15.9 ± 23; [0.08; 90]
	Number of patients with clustered attacks	14 (33%)

Attack duration varied between one and 240 minutes, attack frequency between 0.08 and 90 attacks/month. Attacks reoccurring up to seven days in a “cluster of attacks” were described in 33% of patients.

**Figure 1:**
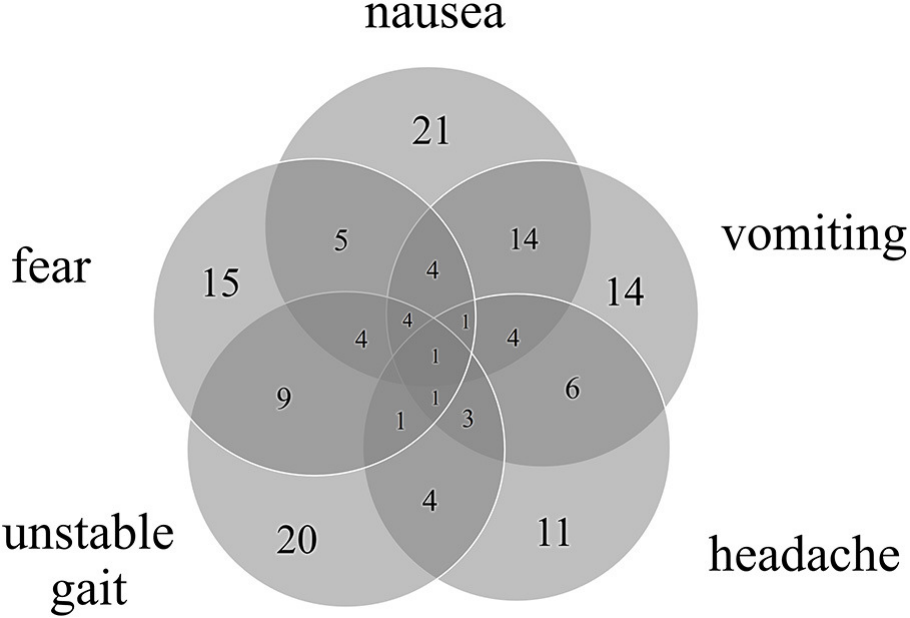
Number of accompanying symptoms and their overlap in 42 patients with RVC. All patients had at least one accompanying symptom, most commonly nausea, unstable gait, and expression of fear.

The most common accompanying symptoms during vertigo attacks were nausea (50%), unstable gait (47%) and expression of fear (Figure 1). Male patients had a significantly higher incidence of vomiting (male = 54%; female = 17%; *p* = 0.047) while non-migrainous headaches tended to be more common in females (male = 16%; female = 39%; *p* = 0.105). The most frequent trigger factors were psychosocial stress (36%) and a systemic or respiratory infection (15%).

### Clinical and instrument-based findings

All patients showed no pathologic findings in the neurological and neuro-otological examinations. In the broad ocular motor examination 12% of patients showed a vertical saccadic smooth pursuit, and 5% an impaired vertical fixation suppression. One child showed an isolated head-shaking nystagmus, two had a slight deviation of the subjective visual vertical, all without any additional evidence of a central or vestibular disorder. The cranial magnetic resonance imaging (MRI) performed in 64% of patients did not reveal any structural pathological findings. Furthermore, all cochlear examinations including AEP's and VEMP's were normal (Table 2).

**Table 2:** Detailed ocular motor and instrument-based findings in the attack-free interval in children with RVC.

**Ocular motor examination**	**% occurrence**
Strabismus	2/42 (5%)
Spontaneous nystagmus in primary position	0/42 (0%)
Gaze-induced nystagmus	0/42 (0%)
Head-shaking nystagmus	1/42 (2%)
Upbeat-/Downbeatnystagmus	0/42 (0%)
Saccadic smooth pursuit movements (horizontal)	0/42 (0%)
Saccadic smooth pursuit movements (vertical)	5/42 (12%)
Impairment of fixation supression (vertical)	2/42 (5%)
Impairment of optokinetic nystagmus (OKN)	0/42 (0%)
Subjective Visual Vertical	2/42 (5%)
Ocular Torsion (under Scanning Laser Ophthalmoscope)	1/42 (2%)
**Instrument-based findings**	
Video-head-impulse-test	0/33 (0%)
Caloric irrigation	1/20 (5%)
Ocular- and cervical evoked potentials	0/21 (0%)
Auditory evoked potentials	0/24 (0%)
Audiometry	0/14 (0%)
Functional sway in posturography	13/25 (52%)
Cranial MRI	0/27 (0%)
EEG	0/24 (0%)
Cardiological examination	0/20 (0%)

The ocular motor examination showed a vertical saccadic smooth pursuit in 12%, an impaired vertical fixation suppression in 5% and a slight deviation of the subjective visual vertical in 5%. The most common finding in the instrument-based diagnostics was a functional sway pattern on posturography. MRI, magnetic resonancy imaging; EEG, Electroencephalography.

### Follow-up

**Figure 2:**
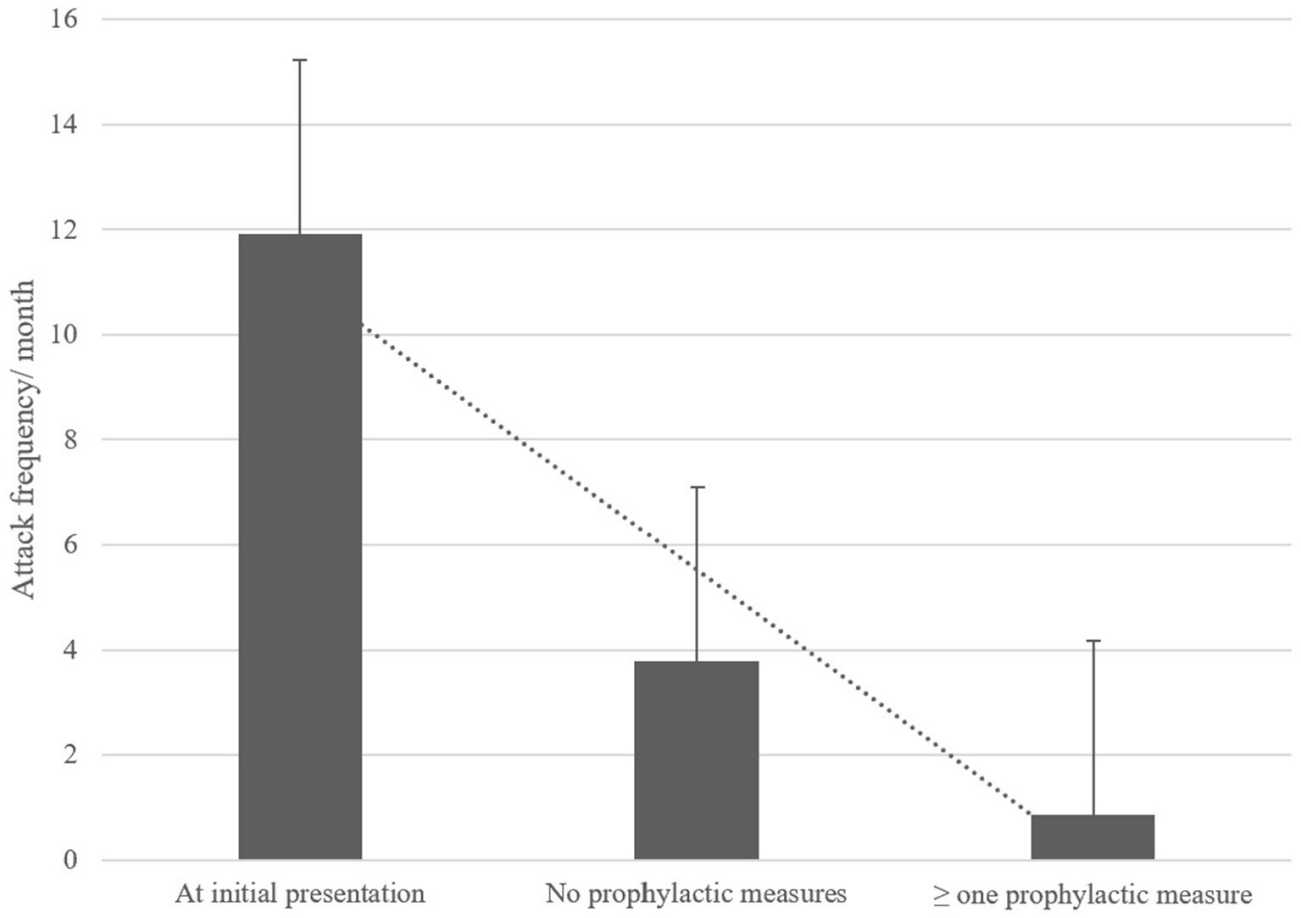
Comparison of attack frequency per month at initial presentation and at follow-up with and without the implementation of prophylactic measures. These measures included regular exercise, increased fluid intake, sleep hygiene, relaxation exercises, daily intake of weight-adapted magnesium. Attacks significantly reduced over time, especially with prophylactic measures.

Twenty-four patients (57%) presented for follow-up examinations and/or completed the follow-up questionnaire. The mean follow-up interval was 3.5 ± 2.6 years. Of the 24 children/adolescents, 13 (54%) still reported suffering from vertigo/dizziness attacks, while in 11 children/adolescents (46%) the attacks had ceased. The mean age of attack cessation was 4.7 ± 2.8 years. In patients still experiencing vertigo attacks the mean attack duration was 41 ± 2.8 min with a frequency of 3.6 ± 5.0 attacks per month. Attack frequency was significantly reduced compared to the initial patient evaluation (initial attack frequency of follow-up patients: 11.9 attacks/month; *p* = 0.038). At follow-up 70% of patients had implemented at least one recommended prophylactic measure, most commonly increased fluid intake and improved sleep hygiene (in 58% of patients). The implementation of measures led to a significantly reduced attack frequency compared to patients that did not conduct any prophylactic measures (0.9 vs. 3.8 per month, *p* = 0.03; see Figure 2), regardless of the implemented measure.

### Migraine association

At initial presentation 11 patients (26%) reported headaches without any migrainous features as an accompanying symptom during vertigo attacks. Five patients (12%) reported only photo-/phonophobia (without headache) during the vertigo attacks. Non-migrainous headaches irrespective of the vertigo attacks were described in 10 patients (23%).

**Figure 3:**
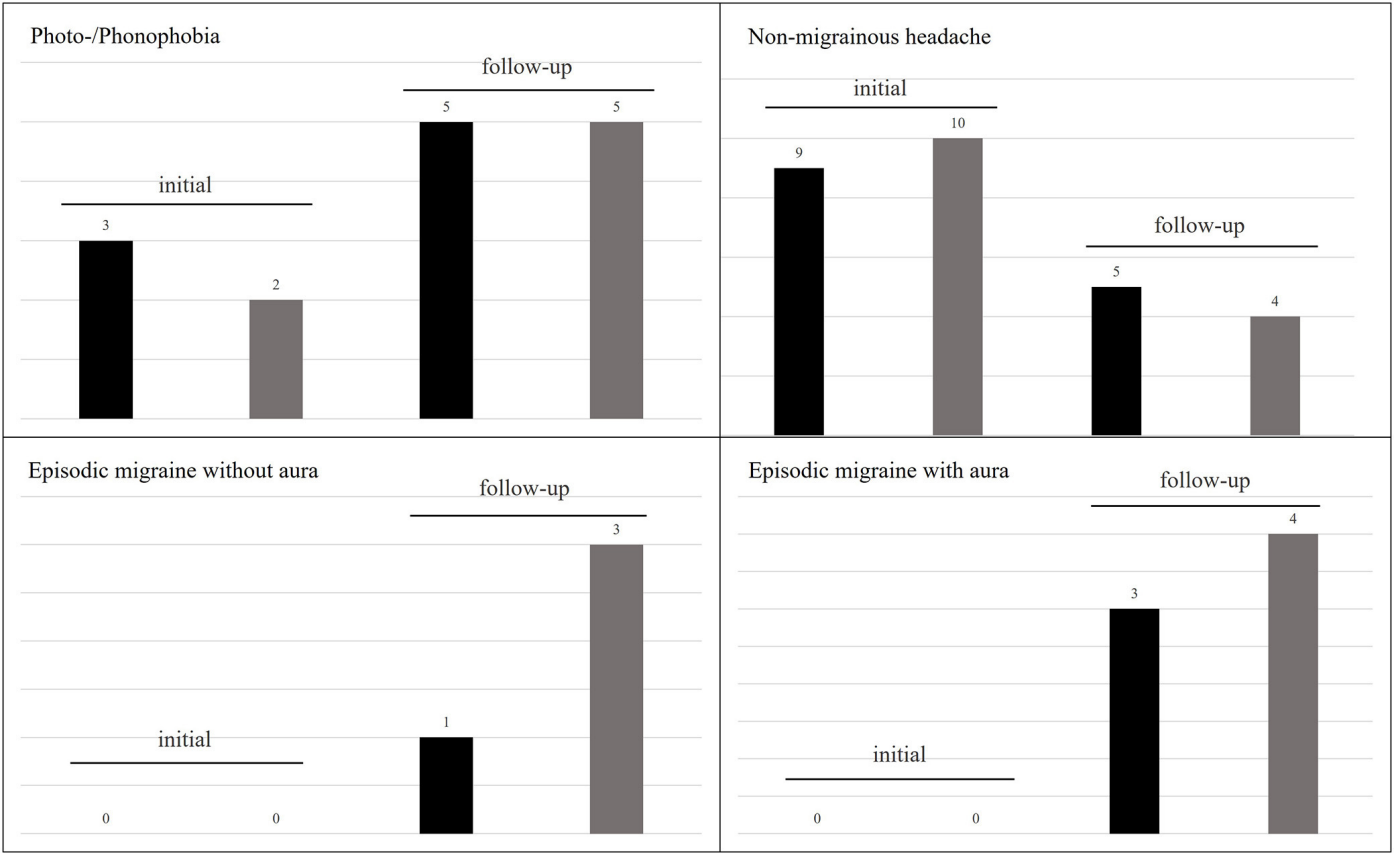
Frequency of headaches and associated symptoms in RVC at initial and follow-up examination. Photo-/phonophobia increased over the course of the disease, while non-migrainous headaches decreased. However, seven patients developed an episodic migraine. The black columns represent patients reporting this symptom during and the gray columns outside of vertigo attacks.

Although initially, none of our patients reported migrainous headaches with photo-/phonophobia or other migraine features during or outside vertigo attacks, at follow-up seven children/adolescents fulfilled the diagnostic criteria for migraine (three with aura, four without aura), of which five still experienced vertigo attacks. In all five cases vertigo attacks were associated to the migraine attacks, fulfilling the diagnostic criteria for VMC. Further details are presented in Figure 3.

Motion sickness as a commonly associated symptom of migraine was only reported in a low number (16%) of all 42 patients. A family history of migraine was positive in 24 patients (57%).

## Discussion

### Clinical characteristics of RVC

In the present cohort of children with RVC, the mean age at first presentation was of 7.1 ± 3.6 years, which is in line with previous findings (15, 18). In most cases, the vertigo/dizziness attacks were described as a torsional or spinning sensation, that lasted 25 min on average and occurred every other day (see Table 1). In the literature, the mean attack duration is similar to the present findings (13, 14), but the reported range varies between very brief attacks (few seconds) (14) and very long attacks (up to 7 days) (13). In our cohort, the shortest attack lasted 1 min and the longest 4 h; the latter considerably differing from previous reports and the diagnostic criteria of the Bárány Society. This disparity is likely due to the fact that 33% of children originally reporting longer attacks (several hours to days), upon more detailed questioning, clearly described brief recurrent vertigo attacks (e.g. few minutes) followed by a vertigo free interval (over several minutes to hours). Such episodes were considered “clustered attacks” (see Table 1) with a high attack frequency and relatively short duration (minutes). We therefore argue that previously described very long RVC attacks may in fact represent a clustering of attacks instead of one attack with the duration of several hours (more than 4–6) or days.

All children reported accompanying symptoms during RVC attacks, most frequently nausea, unstable gait or imbalance, emotional symptoms such as spontaneous crying or expression of fear, vomiting and headache (see Figure 1), consistent with previous findings (13, 14). Notably, 19% of the children included in this study reported falls during the attacks, which has not been previously described. Most of these children also experienced an unstable gait, which might lead to an increased occurrence of falls.

Symptom onset was 2 years later in female patients, attack duration was 30 min longer, and attack frequency more than twice that of male patients. Female patients also had more than twice the incidence of accompanying non-migrainous headaches without phono-/photophobia (male = 16%; female = 39%) at initial presentation. These are to our knowledge the first reports of gender specific differences in RVC. Few gender specific differences in children and adolescents have been reported in headache disorders (19, 20), and vertigo/dizziness complaints (21). Particularly it seems, that the gender specific findings in the present cohort resemble the findings in a pediatric migraine population, that has shown a female predominance in the occurrence of headaches as well as a higher attack frequency with increasing age (20). Whether this finding supports the suggested link of RVC to migraine remains hypothetical.

### Ocular motor and instrument-based findings

In the attack-free interval ocular motor abnormalities were found in a total of 19% of the children with RVC, most frequently a vertical saccadic smooth pursuit and disturbed vertical fixation suppression (see Table 2). We are not aware of any other studies on children with RVC that describe ocular motor findings. Positional nystagmus has previously been found in 20% of children with RVC (15, 22), which we did not find in any examined child; albeit one child had a head-shaking nystagmus with no evidence of any other central or peripheral vestibular imbalance. The observed rate of strabismus is the normal range for children/adolescents (23).

Further instrument-based findings (e.g., MRI, VEMP's, AEP's, etc.) were all normal, except one child showing a pathological side difference in the caloric irrigation. Vestibulo-cochlear symptoms have been described in RVC by Marcelli et al. (15), but caloric irrigation was not conducted. The recently described increased N1-latency and interval of cVEMP's in children with RVC (24) was not observed in the present cohort, although we used standardized VEMP-parameters for the evaluation and did not conduct a further comparison with a healthy, age-matched control group. Overall, slight vestibulo-cochlear deficits such as a pathological caloric irrigation or pathological VEMP findings might be present in a small number of children with RVC and should therefore not lead to the exclusion from the diagnosis of RVC. Furthermore, the examination of balance and postural sway revealed a functional sway pattern (increased postural sway at base-line with “paradoxical” improvement in more demanding conditions) in 52% of patients. This may be indicative of a higher risk of secondary psychosomatic development, as has been described in VMC and migraine-related disorders (25, 26). However, none of the children examined developed a persistent functional disorder over time.

### Long-term follow up and therapeutic approach

Children with RVC commonly show a benign disease course (7, 27–29). Similarly, in the present cohort a cessation of attacks was observed in 44% of patients after a mean follow-up interval of 3.5 years. Furthermore, a significant reduction of attack frequency from an initial mean of 15.9 attacks per month to 3.8 attacks per month was observed at follow-up, suggesting a benign course and spontaneous remission. As a novel finding, the implementation of one or more prophylactic measures (regular exercise, increased fluid intake, sleep hygiene, relaxation exercises, daily intake of weight-adapted magnesium) (see Figure 2), led to a further decrease in attack frequency to 0.9 attacks per month on follow-up, regardless of type and number of implemented measures. These prophylactic measures strongly resemble those applied in children with migraine (30), further underlying the potential causative link between migraine and RVC.

### Headache and migraine

It has been broadly suggested that RVC may be a precursor of migraine (10, 12, 13, 15, 22, 31, 32), although evidence to the contrary also exists (27). While none of our patients reported headaches suggestive of migraine during or in-between vertigo attacks at initial presentation, 40% of patients fulfilled the diagnostic criteria for migraine (38% with, 62% without aura) according to the International Classification of Headache Disorders - ICHD-3 (33) at follow up. Of these, 62% were still experiencing vertigo attacks now accompanied by migrainous headaches, fulfilling the Bárány diagnostic criteria for VMC (6). Overall, our RVC cohort showed a higher prevalence of migraine at the follow-up (after 3.5 ± 2.6 years) than the general population at that age (in children < 14 years: female = 7%; male = 5%) (19). This finding further supports a link of RVC to migraine, although the reason or underlying cause remains unknown.

## Conclusion

In accordance with the present findings from a large cohort of children with RVC, we suggest a more precise characterization of RVC for diagnostic evaluation than suggested by the Bárány-Society. In particular, the age of symptom onset in RVC does not exceed 12 years of age in the present and in any previously published cohort (12, 13, 15, 27, 28). Nevertheless, due to a considerable interval between symptom onset and first evaluation by a physician, the diagnosis of RVC should still be considered in children/adolescents up to 18 years. When evaluating attack duration, a clustering of attacks should be considered, since very long RVC attacks (above 12 h) are rarely mentioned in literature (13) or may be interpreted in the scope of clustered attacks. The presence of slight ocular motor deficits or vestibulo-cochlear dysfunction should not lead to an exclusion of RVC. Furthermore, even though RVC has a benign course, prophylactic measures such as regular exercise, increased fluid intake, sleep hygiene, and relaxation exercises, should be recommended to affected children and their parents.

## Data availability statement

The raw data supporting the conclusions of this article will be made available by the authors, without undue reservation.

## Ethics statement

The studies involving human participants were reviewed and approved by Ethics Committee of the Medical Faculty of the LMU (414-15). Written informed consent to participate in this study was provided by the participants' legal guardian/next of kin.

## Author contributions

KD: data collection, statistical analysis, interpretation of data, conception of tables and figures, and drafting the manuscript. LS: data collection and statistical analysis. EG: data collection, interpretation of data, and revising the manuscript. FF: statistical analysis, interpretation of data, and writing and revising the manuscript. DH: study concept and design, interpretation of data, and revising the manuscript. All authors contributed to the article and approved the submitted version.

## Conflict of interest

The authors declare that the research was conducted in the absence of any commercial or financial relationships that could be construed as a potential conflict of interest.

## Publisher's note

All claims expressed in this article are solely those of the authors and do not necessarily represent those of their affiliated organizations, or those of the publisher, the editors and the reviewers. Any product that may be evaluated in this article, or claim that may be made by its manufacturer, is not guaranteed or endorsed by the publisher.
